# Levels of Soluble CD30 and CD26 and Their Clinical Significance in Patients with Primary Immune Thrombocytopenia

**DOI:** 10.1155/2020/1279371

**Published:** 2020-03-31

**Authors:** Honghui Wang, Xueping Gu, Huiyuan Li, Lingmei Yin, Wei Tao, Jingxing Yu, Qiyuan Zhou, Hongli Mu, Yu Shen, Jin Yao, Lin Liu, Hui Bi, Renchi Yang, Zeping Zhou

**Affiliations:** ^1^Department of Hematology, The Second Affiliated Hospital of Kunming Medical University, Kunming, China; ^2^Guangzhou Women and Children's Medical Center, Guangzhou, China; ^3^State Key Laboratory of Experimental Hematology, Institute of Hematology and Blood Disease Hospital, Chinese Academy of Medical Sciences & Peking Union Medical College, Tianjin, China; ^4^College of Biological Sciences, University of California, Davis, CA, USA

## Abstract

**Background:**

sCD30 and sCD26 are correlated with autoimmune diseases. However, little research has been done on the relationship between them and primary immune thrombocytopenia (ITP).

**Methods:**

This study enrolled 47 patients diagnosed with ITP in the Institute of Hematology and Blood Disease Hospital, Chinese Academy of Medical Sciences (Tianjin, China), from January 2015 to August 2015. The peripheral blood of all subjects was collected. The mRNA expression of CD30 was quantified by RT-PCR, and concentrations of sCD30 and sCD26 were measured by ELISA. Patient characteristics, CD30 mRNA levels, and sCD30 and sCD26 concentrations were analyzed.

**Results:**

The concentration of sCD30 was higher in active ITP patients (median, 35.82 ng/mL) than in remission ITP patients (median, 23.12 ng/mL; *P* = 0.021) and healthy controls (median, 25.11 ng/mL; *P* = 0.002). Plasma sCD26 levels decreased in remission ITP patients compared with that in healthy controls (median, 599.4 ng/mL vs. 964.23 ng/mL; *P* = 0.004). Ratios of sCD26/sCD30 in active ITP patients decreased compared with those in controls (*P* = 0.005). Increased sCD30 was positively correlated with hemorrhage (*r* = 0.493, *P* = 0.017) in ITP patients while little relationship was identified between sCD26 and ITP.

**Conclusion:**

Since sCD30 levels and sCD26/sCD30 ratios may contribute to the activity of the disease, they may be used to assess ITP disease activity.

## 1. Introduction

Primary immune thrombocytopenia (ITP), previously known as idiopathic thrombocytopenic purpura, is an autoimmune hemorrhagic disease. It is caused by autoimmune-mediated platelet destruction and inadequate platelet production by megakaryocyte [1]. The annual incidence of ITP in adults is about 5 to 10 cases per 100,000, with incidence rate higher in females during reproductive age than in males of similar age. People older than 60, regardless of gender, are at higher risk of developing ITP [2]. The most common clinical manifestation of ITP is mucosal and skin hemorrhage, with the most serious being cerebral hemorrhage. Bleeding risk increases along with aging [3]. The first-line therapy against ITP is steroids, and other treatments include intravenous immunoglobulin (IVIg), splenectomy, rituximab, and thrombopoietin receptor agonists (TPO-RA).

In the diagnosis of ITP, key considerations include the patient's medical history, physical examination, complete blood count test, and peripheral blood smear. Additional clues are needed to decide secondary causes of thrombocytopenia due to a lack of diagnostic test which can directly confirm ITP [4].

CD26 and CD30, associated with Th1 and Th2 types of response, respectively [5, 6], release soluble forms of sCD26 and sCD30, into serum after cellular activation. The concentrations of sCD26 and sCD30 increase in some autoimmune disorders such as systemic lupus erythematodes [7], rheumatoid arthritis [8], and ankylosing spondylitis [9]. Thus, sCD26 and sCD30 levels can reflect the activity of these diseases. However, the relationship between sCD26 or sCD30 and ITP, another immune-associated disorder involving the dysfunction of T cells and B cells [10], has hardly been studied. Therefore, we conducted this study to investigate the correlation between them with the hope to help hematologists diagnose ITP, evaluate its activity, and provide therapy.

## 2. Materials and Methods

### 2.1. Patients and Controls

We enrolled 47 patients with ITP (20 males and 27 females), including 23 cases of active ITP and 24 of remission ITP. All patients visited the Institute of Hematology and Blood Disease Hospital, Chinese Academy of Medical Sciences (Tianjin, China), from January 2015 to August 2015. Along with the exclusion of other secondary thrombocytopenia, diagnosis was made with reference to the criteria recommended by the international thrombosis hemostasis working group in 2009 [11]. In addition, 20 healthy persons (10 males and 10 females) participated in our research as controls.

### 2.2. Isolation of Peripheral Blood Mononuclear Cells (PBMCs)

Peripheral blood samples (3-5 mL) were obtained from enrolled individuals using EDTA anticoagulation. Peripheral blood mononuclear cells were isolated by density using the Ficoll-Hypaque gradient centrifugation.

### 2.3. Detection of CD30 mRNA

The total RNA of the isolated PBMCs was isolated by the TRIzol reagent and converted to cDNA by M-MLV RT (Applied Biosystems, USA). The mRNA expression of CD30 was quantified by real-time quantitative PCR (RT-PCR) using SYBR Green (Applied Biosystems, USA) as a double-strand-DNA-specific binding dye on an ABI-7500 Sequence Detection System. RT-PCR primers and sequences are shown in Table 1.

### 2.4. Plasma sCD26 and sCD30 Assays

Plasma samples were stored at −80°C until assayed. Before testing, samples were equilibrated to room temperature. Plasma sCD26 and sCD30 concentrations were measured by enzyme-linked immunosorbent assays (ELISA) commonly used in this area. The lotion and buffer were diluted 20 times with distilled water. ELISA was performed by the sCD30 kit (eBioscience, USA) and sCD26 ELISA kit (eBioscience, USA) according to the manufacturer's instructions. The detection limits were 8.22 ng/mL for sCD30 and 239.22 ng/mL for sCD26. The optical density (OD) values at 450 nm were determined by the ELISA microplate reader (Bio-Rad, USA). The OD value used was the mean value of the duplicate wells. When the value of the duplicate wells was significantly different, the OD value was discarded and remeasured. Concentrations of sCD26 and sCD30 were read from the standard curve generated using the recombinant human sCD26 and sCD30 provided with the assay kit.

## 3. Ethics

The study was approved by the ethics committee of the Second Affiliated Hospital of Kunming Medical University. Written informed consents were obtained before the examination. All methods were performed in accordance with relevant guidelines and regulations.

## 4. Statistical Analysis

Median and range were used for data description. Single-factor variance analysis or *t*-test was used for comparison of sample mean within the group. The Pearson correlation coefficient or Spearman rank correlation was used to analyze the correlation between variables. The results before and after treatment were analyzed by the paired *t*-test. RT-PCR results were analyzed with the relative expression software tool (REST) [12, 13]. *P* < 0.05 was considered statistically significant. All experimental analyses were performed using the GraphPad Prism 5.0 software.

## 5. Results

### 5.1. Plasma Levels of sCD30 and sCD26 in Patients with ITP and Healthy Controls

A total of 47 patients with ITP (20 male and 27 female) participated in this study, with 23 being patients with active disease aged from 18 to 77 years old (mean 46 years old) and 24 being patients in remission aged from 18 to 76 years old (mean 56.5 years old). The control group consists of 20 healthy individuals (10 male and 10 female) aged from 20 to 62 years old (mean 36.5 years old). The baseline characteristics of the patients and controls are summarized in Table 2.

**Figure 1:**
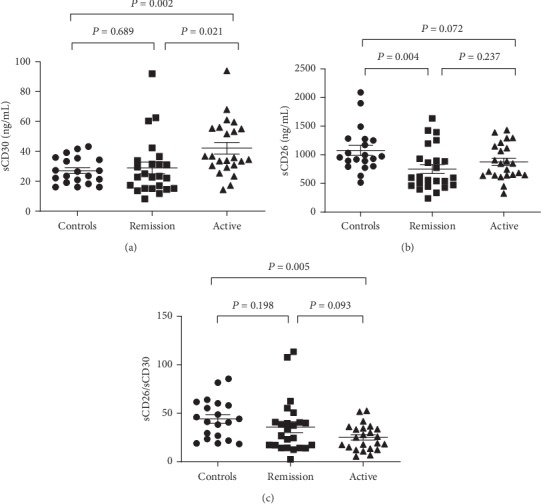
Cytokine levels of the three groups. (a–c) plasma sCD30 and sCD26 levels and sCD26/sCD30 ratios in the controls, the remission group, and the active ITP group, respectively.

Plasma sCD30 levels in the active ITP group (median, 35.82 ng/mL; range, 14.41-93.94 ng/mL) were significantly higher than those in the remission group (median, 23.12 ng/mL; range, 8.22-92.06 ng/mL; *P* = 0.021) and the controls (median, 25.11 ng/mL; range, 16.12-43.28 ng/mL; *P* = 0.002). However, no significant difference of plasma sCD30 levels was found between the controls and the remission group (*P* = 0.689, as shown in Figure 1(a)).

The levels of sCD26 in plasma of the healthy controls (median, 964.23 ng/mL; range, 516.51-2091.38 ng/mL) were significantly higher than those in the remission group (median, 599.4 ng/mL; range, 239.22-1636.09 ng/mL; *P* = 0.004), while no difference was found between the active ITP group (median, 846.01 ng/mL; range, 324.08-1431.99 ng/mL) and controls (*P* = 0.072) and the remission group (*P* = 0.237, as shown in Figure 1(b)).

sCD26/sCD30 ratios in the active ITP group (median, 25.03; range, 5.44-52.53) were significantly lower than those in the controls (median, 42.64; range, 18.37-85.53; *P* = 0.005). There was no difference between the remission group and the active group (*P* = 0.093) or the controls (*P* = 0.198, as shown in Figure 1(c)).

### 5.2. CD30 mRNA Levels in Peripheral Blood Mononuclear Cells (PBMCs)

**Figure 2:**
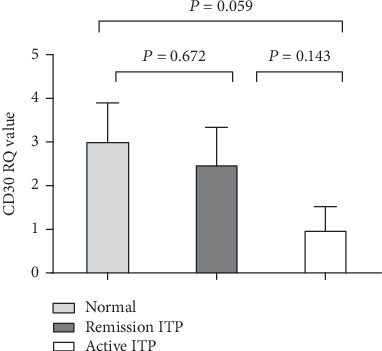
CD30 mRNA levels in peripheral blood mononuclear cells.

RT-PCR showed no significant difference in CD30 mRNA levels among the three groups (active ITP group vs. remission group, *P* = 0.143; active ITP group vs. controls, *P* = 0.059; and controls vs. remission group, *P* = 0.672, as shown in Figure 2).

### 5.3. Relevance of sCD30, sCD26, and sCD26/sCD30 Ratios to Platelet Counts, Disease Courses, and Bleeding in Active ITP Patients

**Figure 3:**
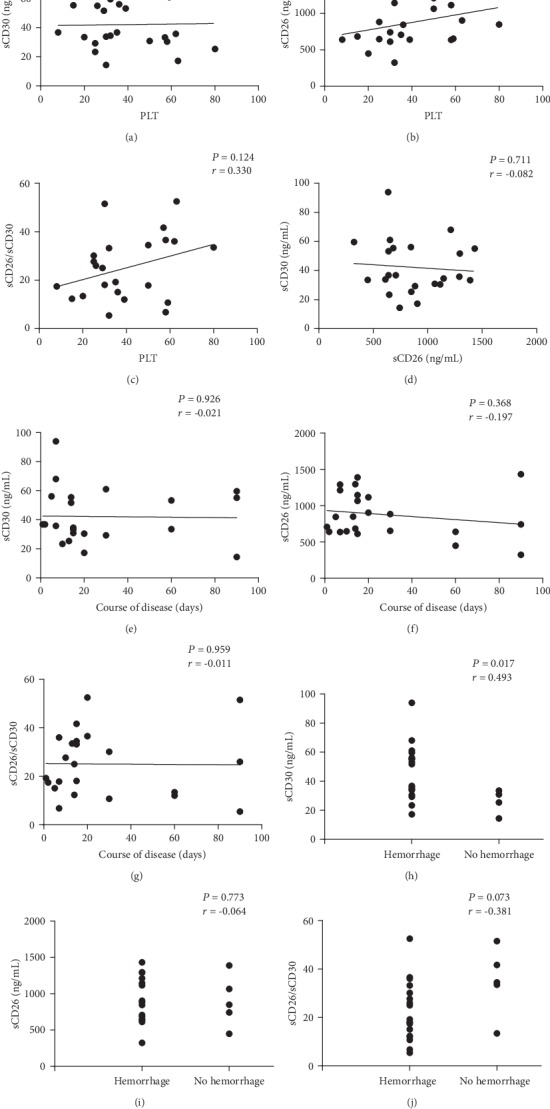
Relevance of the three indicators to platelet counts, disease courses, and bleeding in active ITP patients. (a–c) The relationship of sCD30 levels, sCD26 levels, and sCD26/sCD30 ratios to platelet counts, respectively. (d) The correlation between sCD30 and sCD26. (e–g) The relationship of sCD30 levels, sCD26 levels, and sCD26/sCD30 ratios to disease courses, respectively. (h–j) The relationship of sCD30 levels, sCD26 levels, and sCD26/sCD30 ratios to bleeding, respectively. In (a–g), *r* is Pearson's correlation coefficient while *r* is Spearman's rank correlation coefficient in (h–j). The course of disease is in days.

Platelet counts were not correlated with plasma sCD30 levels, plasma sCD26 levels, and sCD26/sCD30 ratios (*r* = 0.018, 0.304, 0.330, respectively; *P* = 0.936, 0.159, 0.124, respectively; as shown in Figures 3(a)–3(c)). Meanwhile, the concentration of plasma sCD30 was not correlated with sCD26 (*r* = 0.082, *P* = 0.711, as shown in Figure 3(d)) either. Our study of the relationship between the plasma sCD30 levels, sCD26 levels, and sCD26/sCD30 ratios and the disease courses of the patients revealed no statistical difference (*r* = −0.021, −0.197, −0.011, respectively; *P* = 0.926, 0.368, 0.959, respectively; as shown in Figures 3(e)–3(g)). Plasma sCD30 levels were found positively correlated with bleeding (*r* = 0.493, *P* = 0.017) while sCD26 levels or sCD26/sCD30 ratios were not (*r* = −0.064, −0.381, respectively; *P* = 0.773, 0.073, respectively; as shown in Figures 3(h)–3(j)).

### 5.4. Evaluation of Serum Level Changes of Plasma sCD26 and sCD30 after Therapy

**Figure 4:**
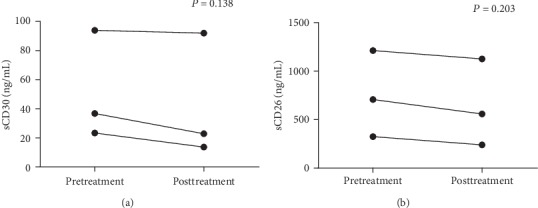
Changes of plasma levels of sCD26 and sCD30 after therapy.

Three active ITP patients without any treatment before recruitment were followed. Significant increased platelet counts were found after therapy (*P* < 0.05). No difference was found in sCD30 or sCD26 after the patients accepted effective treatment (*P* = 0.138, 0.203, respectively, as shown in Figure 4).

## 6. Discussion

CD30, a type I transmembrane receptor (molecular weight 120 kD), is a cell surface molecule belonging to the tumor necrosis factor receptor (TNF-*γ*) superfamily. As a molecular marker of memory T cells in the early stage, CD30 is expressed in activated B cells, particularly in activated Th2 cells [14]. sCD30 is produced by proteolytic cleavage from the surface of CD30+ cells. Elevated sCD30 levels have been found to be associated with many autoimmune diseases. Patients with rheumatoid arthritis have higher levels of serum sCD30 than controls (*P* = 0.008), and the high level of sCD30 is correlated with clinical and laboratory parameters of disease activity [8]. Statistical differences in sCD30 levels have been identified between SLE patients and healthy controls (*P* = 0.0001) as well as between active and nonactive groups (*P* = 0.002) [7]. In this study, we show that the concentration of sCD30 increased in patients with active ITP compared with patients in remission or healthy controls. Meanwhile, sCD26/sCD30 ratios were lower in active ITP patients than in controls. Additionally, plasma sCD30 levels were found positively correlated with bleeding in active ITP patients. Thus, sCD30 may act as a potential indicator to evaluate ITP activity.

Interestingly, rheumatoid arthritis, SLE, and ITP have been regarded as disorders of Th1 cell-polarized immune responses. Serum IFN-*γ* and IL-2, representing the activation of Th1 cells, increase whereas Th2 cytokines (such as IL-4) decrease in ITP [15, 16]. If CD30 is a marker of Th2, it should not increase in Th1 preeminent diseases. Researchers have found increased IL-10, a marker of Th2 cells, in ITP patients, especially in acute patients. This is consistent with the immunotolerance function of IL-10 [17, 18]. Therefore, we speculate that higher sCD30 may be associated with the attempts of Th2 to suppress inflammation [8]. Meanwhile, the CD30 cleavage occurs through a disintegrin and metalloproteinase (ADAM) 10 [19]. Higher expression of ADAM10 has been found in active ITP patients than in healthy controls [20]. Hence, the higher level of plasma sCD30 may result from its potential immunotolerance function and the elevated ADAM10. Since we found no difference in CD30 mRNA between active ITP patients and controls, the increased cleavage rather than increased mRNA levels might have resulted in elevated sCD30 concentrations.

CD26, a multifunctional type II transmembrane glycoprotein with activity of Dipeptidyl Peptidase IV (DDPIV), is highly expressed in Th1 cells and associated with the activation of Th1 cells [21], with sCD26 being the solute form of CD26. Recently, researchers have reported that sCD26 is a negative acute phase protein in many disorders such as rheumatoid arthritis [22], inflammatory bowel disease [23], ANCA-associated vasculitides [24], and SLE [25] due to its decreased concentrations in patients of these diseases. Meanwhile, its weak negative correlation with the activity of the diseases is also reported and the underlying mechanism speculated. Some researchers hypothesize that T cell activation may downregulate the unidentified protease which could cleave and release membrane CD26 and lead to a reduced sCD26 [26]. However, this weak correlation between sCD26 and the diseases is not always verified. For instance, Cuchacovich et al. [27] found that CD26 serum levels in rheumatoid arthritis patients were similar to healthy controls. In our study, we found that sCD26 levels was lower in remission ITP patients than in active ITP patients or healthy control. No difference was observed between active ITP patients and healthy controls. We speculate that the decreased sCD26 levels in remission ITP group may result from different functions of sCD26 in ITP and other immune disorders. Thus, the value of plasma sCD26 levels in ITP remains unclear and requires further research.

One limitation of our study is that the number of patients we followed to detect the changes of sCD30 or sCD26 after they accepted effective treatment was small. Therefore, further multicenter studies with larger sample size are needed.

## 7. Conclusion

In this study, we find higher levels of plasma sCD30 in active ITP patients than in remission patients or healthy controls. sCD30 was positively correlated with the presence of hemorrhage in active ITP patients whereas sCD26 was not. sCD30 concentration and sCD26/sCD30 ratios may be used to assess disease activity. Further research is necessary to determine the precise mechanism.

## Figures and Tables

**Table 1:** RT-PCR primers and sequences.

Gene	Forward sequences (5′⟶3′)	Reversed sequences (5′⟶3′)
CD30	TGCTCTCTGGAGGAAGTGATAG	CAATGTCCAGGTTCTGGTGTAA
*β*-Actin	GGCACCACACCTTCTACAAT	AACATGATCTGGGTCATCTTCTC

**Table 2:** Characteristics of the patients and controls.

	Active ITP	Remission ITP	Controls
Total No. (*n*)	23	24	20
Male/female	8/15	12/12	10/10
Mean age (range, years)	46 (18-77)	56.5 (18-76)	36.5 (20-62)
Mean No. of platelets (range, ×10^9^/L)	35 (8-80)	134 (100-282)	—
Untreated patients	23	1	—
Treated patients	0	23	—
Steroids	—	6	—
Steroids, IVIg	—	3	—
Steroids, danazol	—	7	—
Danazol	—	3	—
Danazol, TPO	—	1	—
Danazol, IVIg, rituximab (MabThera, anti-CD20 MAb)	—	2	—
Steroids, vinblastine	—	1	—

ITP: primary immune thrombocytopenia; IVIg: intravenous immunoglobulin; TPO: thrombopoietin; Danazol: an approved therapeutic modality for ITP.

## Data Availability

The data used to support the findings of this study are available from the corresponding author upon request.

## References

[1] Cines D. B., Blanchette V. S. (2002). Immune thrombocytopenic purpura. *The New England Journal of Medicine*.

[2] Society of Periodontology, CSA (2016). Consensus of Chinese experts on diagnosis and treatment of adult primary immune thrombocytopenia (version 2016). *Zhonghua Xue Ye Xue Za Zhi=Chinese journal of hematology*.

[3] Audia S., Mahevas M., Samson M., Godeau B., Bonnotte B. (2017). Pathogenesis of immune thrombocytopenia. *Autoimmunity Reviews*.

[4] Neunert C., Lim W., Crowther M. (2011). The American Society of Hematology 2011 evidence-based practice guideline for immune thrombocytopenia. *Blood*.

[5] Willheim M., Ebner C., Baier K. (1997). Cell surface characterization of T lymphocytes and allergen-specific T cell clones: correlation of CD26 expression with T (H1) subsets. *The Journal of Allergy and Clinical Immunology*.

[6] Del Prete G., De Carli M., Almerigogna F. (1995). Preferential expression of CD30 by human CD4^+^T cells producing Th2‐type cytokines. *The FASEB Journal*.

[7] Ciferská H., Horák P., Heřmanová Z. (2007). The levels of sCD30 and of sCD40L in a group of patients with systemic lupus erythematodes and their diagnostic value. *Clinical Rheumatology*.

[8] Ulusoy H., Kamanli A., Ilhan N. (2012). Serum levels of soluble CD26 and CD30 and their clinical significance in patients with rheumatoid arthritis. *Rheumatology International*.

[9] Gao R., Sun W., Chen Y., Su Y., Wang C., Dong L. (2015). Elevated serum levels of soluble CD30 in ankylosing spondylitis patients and its association with disease severity-related parameters. *BioMed Research International*.

[10] Giordano P., Cascioli S., Lassandro G. (2016). B-cell hyperfunction in children with immune thrombocytopenic purpura persists after splenectomy. *Pediatric Research*.

[11] Rodeghiero F., Stasi R., Gernsheimer T. (2009). Standardization of terminology, definitions and outcome criteria in immune thrombocytopenic purpura of adults and children: report from an international working group. *Blood*.

[12] Lyu M., Li Y., Hao Y. (2015). Elevated Semaphorin 5A correlated with Th1 polarization in patients with chronic immune thrombocytopenia. *Thrombosis Research*.

[13] Pfaffl M. W., Horgan G. W., Dempfle L. (2002). Relative expression software tool (REST(C)) for group-wise comparison and statistical analysis of relative expression results in real-time PCR. *Nucleic Acids Research*.

[14] Gerli R., Lunardi C., Vinante F., Bistoni O., Pizzolo G., Pitzalis C. (2001). Role of CD30+ T cells in rheumatoid arthritis: a counter-regulatory paradigm for Th1-driven diseases. *Trends in Immunology*.

[15] Ogawara H., Handa H., Morita K. (2003). High Th1/Th2 ratio in patients with chronic idiopathic thrombocytopenic purpura. *European Journal of Haematology*.

[16] Wang T., Zhao H., Ren H. (2005). Type 1 and type 2 T-cell profiles in idiopathic thrombocytopenic purpura. *Haematologica*.

[17] del Vecchio G. C., Giordano P., Tesse R., Piacente L., Altomare M., de Mattia D. (2012). Clinical significance of serum cytokine levels and thrombopoietic markers in childhood idiopathic thrombocytopenic purpura. *Blood Transfusion*.

[18] Cecinati V., Principi N., Brescia L., Giordano P., Esposito S. (2013). Vaccine administration and the development of immune thrombocytopenic purpura in children. *Human Vaccines & Immunotherapeutics*.

[19] Eichenauer D. A., Simhadri V. L., von Strandmann E. P. (2007). ADAM10 inhibition of human CD30 shedding increases specificity of targeted immunotherapy in vitro. *Cancer Research*.

[20] Qiao J., Luo Q., Liu N. (2018). Increased ADAM10 expression in patients with immune thrombocytopenia. *International Immunopharmacology*.

[21] Gorrell M. D., Gysbers V., McCaughan G. W. (2001). CD26: a multifunctional integral membrane and secreted protein of activated lymphocytes. *Scandinavian Journal of Immunology*.

[22] Busso N., Wagtmann N., Herling C. (2005). Circulating CD26 is negatively associated with inflammation in human and experimental arthritis. *The American Journal of Pathology*.

[23] Hildebrandt M., Rose M., Ruter J., Salama A., Monnikes H., Klapp B. F. (2001). Dipeptidyl peptidase IV (DP IV, CD26) in patients with inflammatory bowel disease. *Scandinavian Journal of Gastroenterology*.

[24] Schonermarck U., Csernok E., Trabandt A., Hansen H., Gross W. L. (2000). Circulating cytokines and soluble CD23, CD26 and CD30 in ANCA-associated vasculitides. *Clinical and Experimental Rheumatology*.

[25] Kobayashi H., Hosono O., Mimori T. (2002). Reduction of serum soluble CD26/dipeptidyl peptidase IV enzyme activity and its correlation with disease activity in systemic lupus erythematosus. *The Journal of Rheumatology*.

[26] Sedo A., Duke-Cohan J. S., Balaziova E., Sedova L. R. (2005). Dipeptidyl peptidase IV activity and/or structure homologs: contributing factors in the pathogenesis of rheumatoid arthritis?. *Arthritis Research & Therapy*.

[27] Cuchacovich M., Gatica H., Pizzo S. V., Gonzalez-Gronow M. (2001). Characterization of human serum dipeptidyl peptidase IV (CD26) and analysis of its autoantibodies in patients with rheumatoid arthritis and other autoimmune diseases. *Clinical and Experimental Rheumatology*.

